# Impact of Different Lipid Ligands on the Stability and IgE-Binding Capacity of the Lentil Allergen Len c 3

**DOI:** 10.3390/biom10121668

**Published:** 2020-12-13

**Authors:** Ekaterina I. Finkina, Daria N. Melnikova, Ivan V. Bogdanov, Natalia S. Matveevskaya, Anastasia A. Ignatova, Ilia Y. Toropygin, Tatiana V. Ovchinnikova

**Affiliations:** 1 Science-Educational Center, M.M. Shemyakin and Yu. A. Ovchinnikov Institute of Bioorganic Chemistry, 117997 Moscow, Russia; finkina@mail.ru (E.I.F.); d_n_m_@mail.ru (D.N.M.); contraton@mail.ru (I.V.B.); aignatova_83@mail.ru (A.A.I.); 2 G.N. Gabrichevsky Research Institute of Epidemiology and Microbiology, 125212 Moscow, Russia; matveevskaya@mail.ru; 3 Institute of Biomedical Chemistry, 119121 Moscow, Russia; toropygin@rambler.ru; 4 Department of Bioorganic Chemistry, Lomonosov Moscow State University, 119234 Moscow, Russia; 5 Department of Biotechnology, Sechenov First Moscow State Medical University, 119991 Moscow, Russia

**Keywords:** lipid transfer protein, lentil, allergen, allergenic capacity, lipid ligand, stability, proteolytic digestion, specific IgE

## Abstract

Previously, we isolated the lentil allergen Len c 3, belonging to the class of lipid transfer proteins, cross-reacting with the major peach allergen Pru p 3 and binding lipid ligands. In this work, the allergenic capacity of Len c 3 and effects of different lipid ligands on the protein stability and IgE-binding capacity were investigated. Impacts of pH and heat treating on ligand binding with Len c 3 were also studied. It was shown that the recombinant Len c 3 (rLen c 3) IgE-binding capacity is sensitive to heating and simulating of gastroduodenal digestion. While being heated or digested, the protein showed a considerably lower capacity to bind specific IgE in sera of allergic patients. The presence of lipid ligands increased the thermostability and resistance of rLen c 3 to digestion, but the level of these effects was dependent upon the ligand’s nature. The anionic lysolipid LPPG showed the most pronounced protective effect which correlated well with experimental data on ligand binding. Thus, the Len c 3 stability and allergenic capacity can be retained in the conditions of food heat cooking and gastroduodenal digestion due to the presence of certain lipid ligands.

## 1. Introduction

Today, allergy is one of the most common diseases. About 25–30% of adults and children in different countries suffer from an allergy. Legumes cause many cases of food allergy. Peanut and soybean are at the bottom of the predominant causes of food allergy, but lentil and chickpea also frequently provoke allergic reactions. Lentil is a component of various diets and an important foodstuff of children nutrition. It is mainly eaten after heat cooking, but fresh lentil seedlings are also often used. Lentil is a dietary staple in the Middle East, Asia, Mediterranean area, United States, and Northern Europe [1]. As a consequence, lentil allergy is common in these regions. For example, in Turkey, lentil is reported as the sixth most common food allergen, and in Spain, it is the fourth one causing allergic reactions in pediatric patients [1].

It is known that digestibility of food proteins may determine whether they would be tolerated or become sensitizing agents. High resistance to digestion in the gastrointestinal tract seems to increase a sensitization capacity of proteins. During cooking and digesting in the human gastrointestinal tract, food allergens are exposed to heating and different pH values that can result in denaturation, cleavage, aggregation, and modification of proteins, changing their allergenicity [2]. Recent data show that interaction of food allergens with their ligands leads to structural changes that affect their stability and allergenic properties. Lipid ligands change the process of degradation of lipid-binding food allergens in the human gastrointestinal tract, affect their passage through epithelial barriers, and reduce or increase the efficiency of their interaction with IgE [3,4]. The formation of lipid–protein complex may occur in a natural source of allergen, during cooking or in a human gastrointestinal tract.

Plant lipid transfer proteins (LTPs) are clinically important food allergens which can bind different lipid molecules including fatty acids (FAs), phospho- and galactolipids, sterols, and other ligands [5]. Previously, we isolated from lentil seeds a new LTP, designated as Lc-LTP2 [6], and characterized it as the Len c 3 allergen cross-reacting with the major peach allergen Pru p 3 [7]. We also showed that Len c 3 is able to bind a wide range of lipids, including FAs and lysolipids [8]. Among saturated FAs, the highest binding capacities were observed for lauric acid C12:0 (LAU), while the longer acyl chain FAs arachidic C20:0 (ARA) and behenic C22:0 (BEH) demonstrated a lower affinity for the protein. The highest efficiency of binding was observed for unsaturated FAs with the chain length of 16 and 18 carbon atoms containing from one to three double bonds. The affinity of Len c 3 for anionic lysolipids was higher than that for neutral ones.

In this work, we investigated a susceptibility of the lentil allergen Len c 3 to heat treatment and digestion by enzymes in gastrointestinal tract, as well as effects of various lipid ligands on the protein stability and allergenicity. It is known that legume seeds (including lentil ones) contain a high level of lipids with a profitable composition of unsaturated FAs [9]. The major FAs in lentil seeds are oleic C18:1 (OLE), linoleic C18:2, linolenic C18:3, and palmitic C16:0 (PAL) acids, but such acids as LAU, stearic C18:0 (STE), ARA, and BEH are also present there [9,10]. Along with FAs, lentil seeds contain neutral lipids, glycolipids, and different phospholipids [11]. The main components of bile, secreted into the intestinal lumen, are cholesterol, bile acids, bilirubin, and phospholipids, mainly phosphatidylcholine and lysophosphatidylcholine composed FAs such as PAL, OLE, and linoleic [12]. Therefore, in order to study effects of possible endogenous and exogenous ligands on the stability and allergenic properties of Len c 3, FAs (LAU, STE, BEH, and OLE), and lysolipids (lyso-palmitoyl phosphatidylcholine (LPPC) and lyso-palmitoyl phosphatidylglycerol (LPPG)) were used.

## 2. Materials and Methods

### 2.1. Materials

Synthetic lysophospholipids were purchased from Avanti Polar Lipids (Alabaster, AL, USA). FAs and 2-*p*-toluidinonaphthalene-6-sulphonate (TNS) were purchased from Sigma-Aldrich (St. Louis, MO, USA). In all experiments, the recombinant Len c 3 (rLen c 3) and Pru p 3 (rPru p 3) overexpressed in *Escherichia coli* and purified as described previously [6,7,13] were used (see Supplementary Materials). The identity of natural and recombinant proteins was shown by us previously [6,7,13].

### 2.2. Mass Spectrometry

All mass spectrometry measurements were performed by using Ultraflex III MALDI-TOF mass spectrometer (Bruker, Bremen, Germany) equipped with a UV Nd:YAG laser (355 nm) and LIFT MS/MS unit. The molecular weights of the recombinant LTP and digestion products were determined in a linear or reflector mode. Amino acid sequences of peptide hydrolysis products were confirmed by using MALDI MS/MS analysis. Disulfide bonds were reduced with dithiothreitol.

### 2.3. Circular Dichroism Spectroscopy

Circular dichroism spectra were recorded at different temperatures, using a J-810 spectropolarimeter (Jasco, Hachioji, Tokyo, Japan) in a 0.1 cm path length quartz cell (Hellma GmbH & Co. KG, Mullheim, Germany) in the 190–250 nm range, and 0.2, using solutions of rLen c 3 in 10 mM phosphate buffer, pH 7.4, or 150 mM sodium chloride, pH 2.5, simulated gastric fluid, at a concentration of 17 µM. Fatty acids or lysolipids were added at a final concentration of 1 mM. Each experiment was performed twice, independently.

### 2.4. Lipid Binding

The ability of rLen c 3 to bind lipids was assayed by monitoring the displacement of the fluorescent probe TNS. Fluorescence intensity was measured at 25 °C with a F-2710 spectrofluorimeter (Hitachi, Tokyo, Japan), using a 10 mm slit width for both excitation (λ = 320 nm) and emission (λ = 437 nm) and the measurement taken for no longer than 1.5–2 s. After equilibrating TNS (4 μM) in 10 mM phosphate buffer, pH 7.4, or 150 mM sodium chloride, pH 2.5, for 2 min with gentle mixing, the 2 mM of competing ligand was titrated into the 2 mL of 4 μM rLen c 3 solution in 1 μL aliquots. The resulting data were fitted by using a sigmoidal curve-fitting in GraphPad Prism from which the concentration able to displace 50% of the TNS (IC_50_) was calculated. The dependence of the ligand binding on temperature was performed at 20, 40, 60, 90, and 20 °C, after cooling down as previously described [14]. Temperature was checked with a temperature probe in the cuvette. Each experiment was performed in triplicate, independently.

### 2.5. In Vitro Gastrointestinal Digestion

Simulation of gastrointestinal digestion in vitro was performed as described in [15]. Briefly, gastric digestion was performed for 2 h, using 50 ng of pepsin (*p*) (Sigma) per 1 μg of rLen c 3 in 0.1 M HCl, pH 2.0 (final protein concentration 0.05 mM). For duodenal digestion, pH of the mixture resulting from gastric digestion was adjusted to 8.0 by addition of ammonium bicarbonate. The obtained mixture was incubated for 24 h at 37 °C with 2.5 ng of trypsin (t) (Promega, Madison, WI, USA) and 10 ng of α-chymotrypsin (ch) (Sigma) per 1 μg of the substrate. Degree of proteolysis was monitored by sodium dodecyl sulfate polyacrylamide gel electrophoresis (SDS-PAGE) under reducing conditions [16] and reversed-phase high-performance liquid chromatography (RP-HPLC) on Luna C_18_ column (5 μm, 250 × 4.6 mm; Phenomenex, Torrance, CA, USA) at a flow rate of 0.5 mL/min, using a gradient of acetonitrile concentration from 5 to 65% for 45 min in 0.1% trifluoroacetic acid (TFA). Gel analysis was performed, using a densitometer. Electroblotting and subsequent N-terminal protein sequencing were performed as described [13]. The protein was preheated at 100 °C for 30 min to study the effect of temperature on allergen stability. rLen c 3 was preloaded with various lipid ligands (LTP to lipid molar ratio 1:4) for 10 min, in order to investigate their effects on allergen resistance to proteolytic digest. As a control of the influence of lipid ligands on the work of proteolytic enzymes, the same reactions were carried out, using bovine α-casein (Sigma) as a substrate. Some independent experiments were performed in each case.

### 2.6. Patients’ Sera and Immunoglobulin Binding Assay

Sera from allergic patients (*n* = 100) with food and pollen allergy were obtained from the Clinical Diagnostic Center of G.N. Gabrichevsky Research Institute for Epidemiology and Microbiology. The work with human sera was approved by the local ethical committees of this Institute (FS-99-01-009026, Moscow, Russia). The amount of specific IgE (sIgE) to allergen extracts in the patient sera was determined by using RIDA qLine Allergy Panel 1-4 (R-Biopharm, Pfungstadt, Germany). Screening of sera containing sIgE to peach Pru p 3 and Len c 3 was performed by ELISA. For that, plate wells (Corning Incorporated, Corning, NY, USA) were coated with recombinant allergen (0.5 μg/well) in 0.01 M phosphate buffered saline (PBS), pH 7.4, for 1 h, at 37 °C; saturated with 2% bovine serum albumin (SERVA, Heidelberg, Germany) in PBS buffer for 1 h, at 37 °C; and then incubated with the sera of allergic patients (1:4 dilution), for 2 h, at 37 °C. Specific IgE binding was detected by using peroxidase-conjugated anti-human IgE from goat (Sigma) (1:2000 dilution), and 3,3′,5,5′-tetramethylbenzidine (TMB) liquid substrate system for ELISA (Sigma). The enzymatic reaction was stopped after 30 min by 2 N H_2_SO_4_ and absorbance values were determined at 450 nm. Moreover, 0.01 M PBS with 0.05% Tween-20 was used as a washing solution on each step. For sIgE binding experiments with the intact or heated (at 100 °C for 30 min) or digested (gastric and subsequent duodenal digestion for 2 h) rLen c 3, patient sera containing sIgE were selected (Table S3). Intact and treated rLen c 3 samples were coated in wells of 96-well plates (0.5 μg/well), and ELISA was performed as described above. Sera samples from non-allergic individuals were used as a negative control.

To study the effects of lipid ligands on IgE binding capacity of allergen, rLen c 3 was covalently bound to Nunc Immobilizer Amino surface plates (Thermo Scientific, Waltham, MA, USA), according to peptide coupling protocol. rLen c 3 solution (100 μg/mL) in 100 mM sodium carbonate, pH 9.6, was added to the wells; after that, the plates were incubated at 25 °C for 2 h. Then 10 mM ethanolamine at the same buffer was used for the plates post-coupling at 25 °C for 1 h. Pre-incubation with PBS or lipid ligand solutions in PBS at concentration of 60 μM was performed overnight at 4 °C. After that, the plates were incubated with selected sera in 1:4 dilution, overnight, at 4 °C, with the absence or presence of 10 μM lipid ligands, due to the reversibility of Len c 3-ligand complexes. Subsequent steps of ELISA were carried out as described above. This experiment was performed twice, independently.

## 3. Results

### 3.1. Effects of pH Change and Heating on Ligand Binding to rLen c 3

To study an influence of different pH values and heating on ligand binding capacity of the lentil allergen Len c 3, TNS displacement assay was used. TNS is a hydrophobic fluorescence probe that is highly fluorescent when bound to the hydrophobic cavity of the protein and competed with lipid molecules for binding with Len c 3. No quenching resulting from the interaction between each of the selected ligands (FAs and lysolipids) and TNS alone was detected, showing that these molecules did not interact directly. 

The rLen c 3 affinity to the tested ligands were measured in 10 mM phosphate buffer, pH 7.4, and in 150 mM sodium chloride buffer, pH 2.5, mimicking gastric juice. At an acidic pH value, the affinity for LPPC slightly increased; for LPPG and LAU, it remained unchanged, but for STE and OLE, it decreased (Table 1).

**Figure 1:**
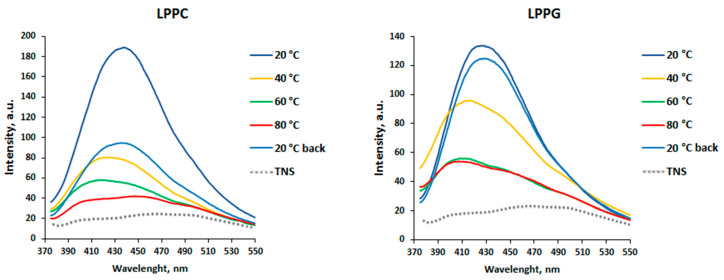
TNS–rLen c 3 fluorescence spectra at pH 7.4, at temperature values ranging from 20 °C (dark blue line) to 90 °C (red line), and at 20 °C after cooling down (blue line). The spectrum of TNS alone is shown as a reference (dashed line).

An ability of rLen c 3 to bind LPPC and LPPG having different charge and the structure of the polar head at various temperature values at pH 7.4 was investigated (Figure 1). As far as the temperature was increased from 20 to 90 °C, the fluorescence emission intensity decreased. Then the samples were cooled down to 20 °C, and the fluorescence emission intensity was shown to be increased in varying degrees. A gradual decrease in the fluorescence emission intensity was observed upon binding of LPPC to rLen c 3. When the sample was cooled down to 20 °C, the ability of rLen c 3 to bind LPPC was not restored. In the case of LPPG binding at temperature values from 40 to 90 °C, data revealed a blue-shift of the fluorescence maximum wavelength and a sharp drop in the signal intensity. It is interesting to note that the ability of rLen c 3 to bind LPPG was almost completely restored after the sample was cooled down to 20 °C.

### 3.2. Effects of pH and Heating on the Secondary Structure of rLen c 3

**Figure 2:**
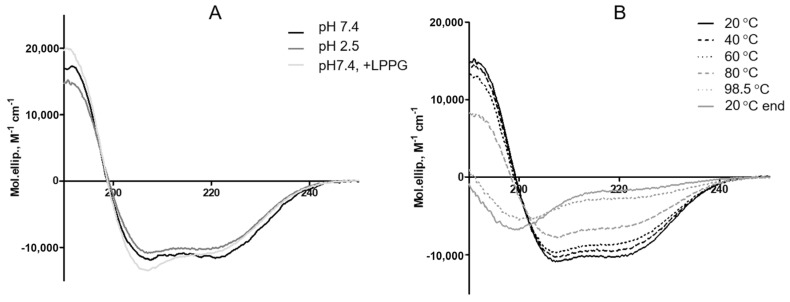
Circular dichroism spectra of rLen c 3. (**A**) Overlay of the spectra of the liganded and unliganded protein at pH 7.4 and of the unliganded protein at pH 2.5 (all measured at 20 °C). (**B**) Effects of heating up to 98.5 °C and subsequent cooling down to 20 °C on the secondary structure of rLen c 3 at pH 7.4.

The pH and heat stability of rLen c 3 were examined by CD spectroscopy. The CD spectrum of rLen c 3 at pH 7.4 showed predominantly α-helical structure with typical negative extremes at 208 and 222 nm and a positive maximum at 195 nm. At an acidic pH value mimicking gastric juice, the protein CD spectrum remains almost unchanged (Figure 2A). Under acidic conditions, the percentage of α-helices increased slightly. As recently shown by us [17], the temperature values above 80 °C were required to lead to the allergen denaturation (Figure 2B and Table S1). At 98.5 °C, the protein became almost completely unfolded. After cooling down to 20 °C, the CD spectrum was not recovered.

### 3.3. Effects of Different Lipid Ligands on Thermostability of rLen c 3

**Figure 3:**
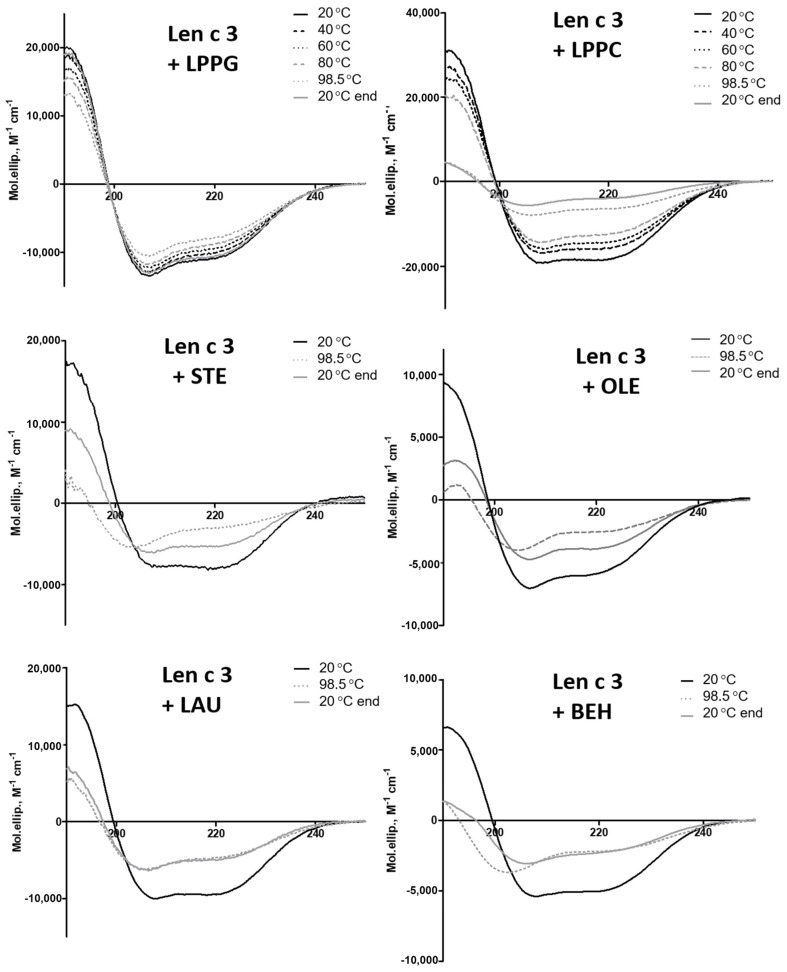
Effect of heating up to 98.5 °C and subsequent cooling down to 20 °C on the secondary structure of rLen c 3, measured at pH 7.4, in the presence of different lipid ligands.

Circular dichroism spectroscopy was also used for investigation of an influence of the selected lipid ligands (FAs and lysolipids) on thermostability of rLen c 3. The presence of lipid ligands did not lead to significant changes in the secondary structure of the allergen at pH 7.4, at 20 °C, with the exception of LPPC, which increased the percentage of α-helices in rLen c 3 (Figure 2A and Table S1). It was found that lipid ligands affected a resistance of the protein to thermal denaturation in varying degrees (Figure 3 and Table S1). The most pronounced effect on the rLen c 3 thermostability was observed in the presence of LPPG. Heating even up to 98.5 °C practically did not change the protein structure in the presence of LPPG, and subsequent cooling down to 20 °C led to complete recovery of the native conformation. In the presence of LPPC, a complete denaturation of the protein did not occur even at 98.5 °C, as it took place in the case of an unliganded protein, but recovery of the protein structure was not observed after cooling down. STE and OLE increased the rLen c 3 thermostability, but the effect was less pronounced than in the case of LPPG. After cooling down, a partial protein renaturation occurred, and the content of α-helices increased in the presence of STE and OLE. On the contrary, LAU and BEH had a slight protective effect on the protein structure.

### 3.4. Effects of Different Lipid Ligands on Gastrointestinal Digestion of rLen c 3

**Figure 4:**
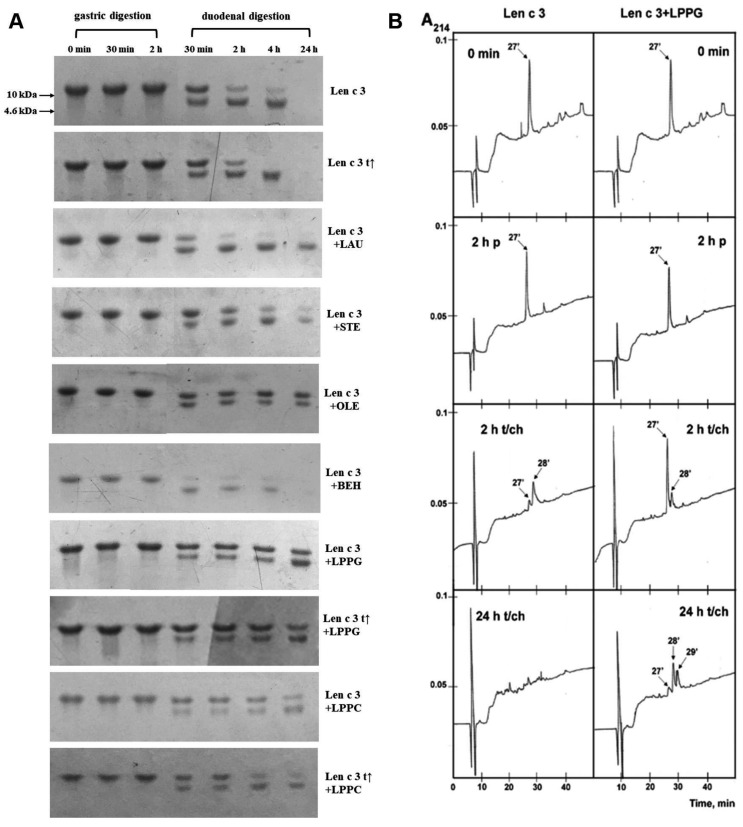
Effect of heating (t↑) and the presence of different lipid ligands on mimicked gastrointestinal digestion of rLen c 3, as detected by means of SDS-PAGE (**A**) and RP-HPLC (**B**) on Luna C_18_ column with a linear gradient of acetonitrile concentration from 5% to 65% for 45 min (0 min, 2 h p—gastric digestion, 2 h and 24 h t/ch—subsequent duodenal digestion).

The lentil allergen rLen c 3 showed a remarkable stability to gastric digestion. Only one evident band, corresponding to the intact rLen c 3, was revealed by SDS-PAGE, even after 2 h of digestion (Figure 4A). At the same time, bovine α-casein, which was taken as a control for comparison, almost completely degraded by pepsin in the first 5 min. Subsequent treatment mimicking duodenal digestion resulted in the effective rLen c 3 degradation, with appearance of the digestion product with molecular mass of ~8 kDa, as shown by SDS-PAGE (Figure 4A). According to densitometric gel analysis, more than 65% and 85% of the intact protein was cleaved after 30 min and 2 h of the protein duodenal digestion, respectively (Figure S1). After 24 h of the duodenal digestion, 100% rLen c 3 degradation was observed, including cleavage of its stable proteolytic 8-kDa fragment. The protein preheating at 100 °C for 30 min before digestion slightly increased an efficiency of the allergen degradation. Transfer of the 8 kDa proteolytic fragment onto the Immobilon-P PVDF membrane and its N-terminal sequencing yielded the amino acid sequence AISXGAVTSDL, which completely matched the first 11 residues of the Len c 3 primary structure. Therefore, it was shown that, during gastrointestinal digestion, primarily the degradation of the C-terminal part of the protein occurred.

The presence of the selected lipid ligands affected the allergen resistance to proteolytic digestion (Figure 4A and Figure S1). All ligands except BEH reduced the protein degradation rate, and this effect was more pronounced in the presence of LPPG and OLE. Only ~55% degradation of rLen c 3 occurred even after 24 h of duodenal digestion in the presence of these ligands. STE and, to a lesser extent, LPPC slowed down the allergen degradation. In the presence of LAU, the rLen c 3 allergen (but not its 8 kDa fragment) was quickly digested by trypsin/chymotrypsin. Interestingly, the presence of BEH even slightly accelerated the allergen proteolysis. The proteolytic 8 kDa fragment was more stable than the intact protein and remained in the reaction mixture, even after 24 h, in the presence of all ligands except BEH. The protein preheating before digestion in the presence of LPPG and LPPC slightly increased the rate of the allergen degradation after 4 and 24 h, and this effect was more pronounced in the case of LPPC.

The results of gastroduodenal digestion of rLen c 3 alone and in the presence of LPPG were also analyzed by RP-HPLC (Figure 4B). The only peak in the chromatograms at 27 min, corresponding to the intact allergen, was detected after gastric digestion. After 2 h of duodenal digestion, an additional peak at 28 min corresponding to the 8 kDa proteolytic product was detected. The 8 kDa peak was much smaller in the case of the protein proteolysis in the presence of LPPG. After 24 h of duodenal digestion, there were no peaks in the case of digestion of rLen c 3 alone, while in the presence of LPPG, the peaks at 27, 28, and 29 min were observed. Mass spectrometry analysis revealed the peak at 27 min in all the samples (0 min; 2 h p—gastric digestion; and 2 h and 24 h t/ch—subsequent duodenal digestion), which corresponded to Len c 3 stabilized by four disulfide bonds ([M+H]^+^ *m*/*z* 9282.8 and [M+2H]^2+^ *m*/*z* 4641.8) (Supplementary Materials Figure S2). Apparently, the disulfide-stabilized protein with a cleavage of one internal peptide bond (*m*/*z* 9302.5), the fragment without the C-terminal Phe93 residue (*m*/*z* 9137.1), and the protein fragment 1–80 (*m*/*z* 7846.3, 3 SS) were present in the peak at 28 min in the samples after duodenal digestion. Additional low-molecular-mass peptides and the protein fragment with *m*/*z* 9173.4 were detected in the peak at 29 min. The last one is most likely the disulfide stabilized protein without one internal Lys (34, 53, or 81), as well as with one cleaved internal peptide bond. The reduction by dithiothreitol of the fragment contained in the fraction at 28 min led to a decrease in the intensity of the peak with *m*/*z* 9302.5 and an increase in intensity of the peak corresponding to the protein fragment 1–80 (*m*/*z* 7853.7), wherein no peaks with other molecular masses were detected in a linear mode. Possibly, in the disulfide-stabilized fragment with *m*/*z* 9302.5 the peptide bond after Tyr80 was cleaved. These results indicated that the C-terminal part of Len c 3 molecule was most sensitive to gastroduodenal digestion, and the 8 kDa proteolytic product detected by SDS-PAGE most likely corresponded to the protein fragment 1-80.

**Figure 5:**
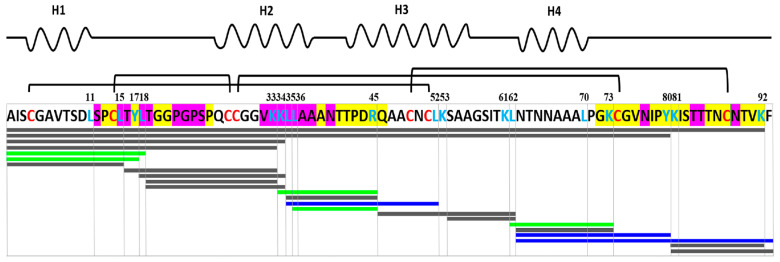
Peptide profiling of rLen c 3 gastric and subsequent duodenal digestion for 2 h. Peptides identified by mass spectrometry are shown as blue (rLen c 3 alone), green (rLen c 3 with LPPG), and gray (in both cases) bars. The key amino acid residues identified as cleavage sites are marked in blue. The residues forming three linear epitopes of Pru p 3 are colored in yellow (matching) and magenta (not matching). Location of four α-helices H1–H4 (PDB ID: 5LQV) is shown at the top of the figure.

Mass spectrometry of total hydrolysates after gastroduodenal digestion of rLen c 3 alone and in the presence of LPPG was carried out by using MS/MS analysis of fragments (Figure 5 and Table S2). After 2 h of duodenal digestion, 19 and 21 fragments of the protein were identified on the MALDI spectra of Len c 3, alone and in the presence of LPPG, respectively. There was no significant difference between the results of digestion in the presence or absence of the lipid. Along with that, the intact rLen c 3, its large fragment 1-80, and the allergen with a cleavage of one internal peptide bond or without the C-terminal Phe93 residue were found among the RP-HPLC fractions. Besides peptide bonds after Tyr80 and Lys92 residues, rLen c 3 was efficiently cleaved at the peptide bonds formed by carboxyl groups of Lys33, Lys34, and Leu62. The number of peptide fragments formed as a result of cleavage of the peptide bond after Arg45 or Leu62 was, more or less, respectively, in the presence of LPPG. Cleavage of the peptide bonds formed by carboxyl groups of Leu11, Leu36, Leu70, and Lys81 was not observed in both cases. The disulfide-stabilized protein fragments 1-33 (*m*/*z* 3154.8) and 1-34 (*m*/*z* 3283.7) with a cleavage of one peptide bond were identified in both digests. This was confirmed by reduction of digests with dithiothreitol, where after both fragments were not observed in the mass spectra.

### 3.5. Effect of Gastric Conditions and Heat Treatment on IgE-Binding Capacity of rLen c 3

**Figure 6:**
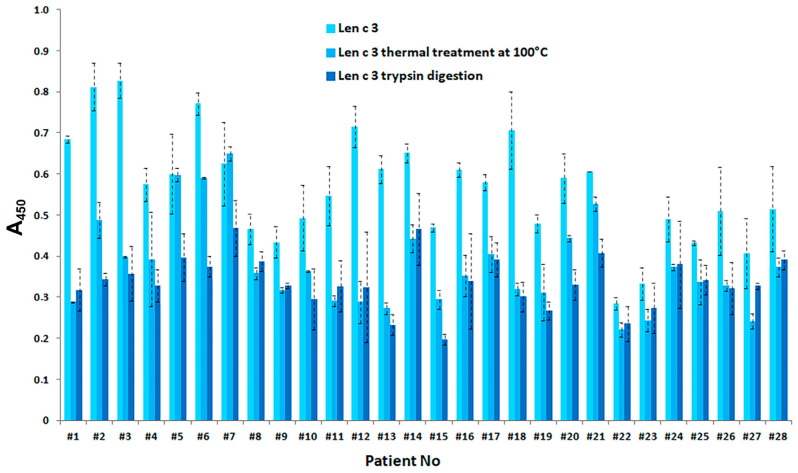
ELISA with intact, heated, and digested rLen c 3, using the patient sera containing sIgE to this allergen. Differences in mean A values were pair-wise compared by a Wilcoxon signed-rank test (W-test). In both groups, either after thermal treatment or after trypsin digestion, the *p*-value was <0.001, compared to A values for the untreated rLen c 3.

In order to evaluate IgE-binding capacity of rLen c 3 after heat treatment at 100 °C for 30 min, as well as after gastric and subsequent duodenal digestion for 2 h, ELISA method was used. It was shown that a number of LTP-related allergies, including the lentil allergy, are mediated by sensitization to the major peach allergen Pru p 3. Earlier, we demonstrated that the lentil allergen Len c 3 is cross-reactive with the major sensitizer of LTP family Pru p 3 [7]. Therefore, sera from patients with food and pollen allergy were screened for the presence of specific anti-rPru p 3 IgE at the initial step; after that, selected sera were examined for the cross-reactivity of sIgE to rLen c 3 (Table S3). For all rPru p 3 IgE^+^ sera, we observed IgE-binding with rLen c 3, but with lower values than for rPru p 3 in all cases. We showed that rLen c 3 significantly (*p* < 0.001) reduced its IgE-binding capacity after heating and proteolytic digestion (Figure 6).

### 3.6. Effects of Different Lipid Ligands on IgE Binding Capacity of rLen c 3

**Figure 7:**
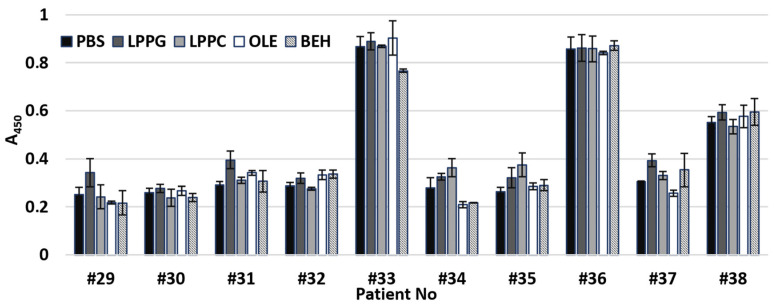
ELISA with ligand-free rLen c 3 or with rLen c 3 preincubated with lipid ligands. The patient sera containing sIgE to this allergen were used. Differences in mean A values were compared by a Wilcoxon signed-rank test (W-test). No significant difference in IgE binding to rLen c 3 alone or to the allergen after its preincubation with different lipid ligands was observed (*p* value > 0.1).

To investigate an influence of different lipid ligands on IgE binding capacity of rLen c 3, sera from 10 patients with food and pollen allergy containing specific IgE were also used (Table S3). No significant difference in IgE binding to rLen c 3 alone or to the allergen after its preincubation with different lipid ligands was observed (Figure 7). Nevertheless, for some sera, a small increase of IgE binding capacity of rLen c 3 was observed in the presence of LPPG; however, this increase was also insignificant (*p* > 0.1).

## 4. Discussion

Many research groups all over the world are working in the field of molecular allergology, to gain insight into the sensitization process by food and inhalant allergens. Recently, it has been shown for legumes that a protein allergenicity does not always correlate with frequency of consumption of a legume-containing product, as well as with its quantitative content in the legume. Other factors, including allergen physicochemical properties, its processing, effects of different allergen ligands, and a patient’s individuality, may play an important role in the prevalence of sensitization [18]. Lentil is an increasingly consumed food product in many countries, and the lentil allergy has a great importance, especially among children, due to high frequency of anaphylactic reactions [19].

Previously, we isolated and characterized the lentil allergen Len c 3, belonging to LTP class [7,20]. In this paper, we characterized Len c 3 properties in more details and study effects of different lipids ligands on stability and IgE-binding capacity of this protein. First, pH and thermal stability of the lentil allergen rLen c 3 were examined. As we showed earlier, the spatial structure of Len c 3 is similar to that of other plant lipid transfer proteins and consists of four α-helices and a long C-terminal tail without a regular secondary structure [6]. CD spectra of rLen c 3 at pH 7.4 and 2.5 were identical and had the shape characteristic for α-helical structure. This was indicated that the lentil allergen was stable under acidic conditions and did not denature in a human stomach. On the other hand, the protein denaturation occurred upon heating from 80 to 98.5 °C, and its structure did not restore after cooling down (Figure 2) [17]. Previously, similar results have also been obtained for the peach Pru p 3 and cherry Pru av 3 allergens [21,22]. It is important to note that, that heated for 30 min at 100 °C rLen c 3 showed a remarkably lower capacity to bind sIgE from sera of allergic patients (Figure 6), which is likely associated with unfolding of conformational epitopes of the allergen.

Then, effects of pH and heating on the rLen c 3 capacity to bind different endo- and exogenic lipid ligands, including FAs and lysolipids, were studied. An acidic pH had almost no effect on the protein ability to bind various ligands (Table 1). However, rLen c 3, heated from 20 to 90 °C, reduced its ability to bind lysolipids LPPC and LPPG (Figure 1). However, this effect was more pronounced in the case of the neutral lipid LPPC, which was bound by the protein with lower efficiency. Moreover, the rLen c 3 ability to bind negatively charged LPPG was restored after cooling down to 20 °C. Probably, in the presence of LPPG, the protein denaturation upon heating occurred to a lesser extent.

Next, an influence of lipid ligands on heat sensitivity of rLen c 3 was investigated. The presence of FAs and lysolipids increased heat stability of rLen c 3 at neutral pH, but this effect was expressed to varying degrees. Among all ligands, the most pronounced protective effect on the protein structure had LPPG (Figure 3 and Table S1). At the same time, LPPC increased rLen c 3 thermostability much weaker than LPPG. In the presence of LPPG, the allergen was stable and completely retained its structure upon heating up to 98.5 °C. This was in good agreement with obtained data on LPPG binding upon heating described above. Among FAs, OLE showed the most pronounced protective effect on the protein structure. In the presence of STE, the degree of rLen c 3 denaturation upon heating was higher. An imperceptible effect was observed for rLen c 3 binding with BEH which practically did not interact with each other due to the size restrictions of the protein hydrophobic cavity [7]. It was assumed that LPPG less efficiently displaced TNS from the protein cavity than LAU and OLE having a smaller size, but formed more stable complex with the lentil allergen. As a consequence, LPPG demonstrated more pronounced protective effect.

Sensitivity of rLen c 3 to gastroduodenal digestion was also examined. rLen c 3 like LTP from peach [23] was not sensitive to gastric digestion due the fact that leucine residues in its structure were mainly located in the hydrophobic cavity of the protein (PDB ID: 2MAL) and were inaccessible to pepsin (Figure 4 and Supplementary Materials Figure S1). At the same time, rLen c 3 was cleaved efficiently even after 2 h of subsequent duodenal digestion. Preheating of the lentil allergen did not increase notably the rate of rLen c 3 proteolysis although the protein denaturation took place as it was shown by CD spectroscopy. Possibly, upon heating disulfide bonds playing a critical role in stabilization of LTP structure remained unbroken. Their reduction led to a significant increase in the rate of tryptic digestion of rLen c 3 as we showed earlier [20]. Preservation of disulfide bonds might also explain an ability of the protein heated to 90 °C to bind LPPG and, to a lesser extent, LPPC (Figure 1). Duodenal digestion at the initial stage resulted in the cleavage of the rLen c 3 labile C-terminal part lacking a regular secondary structure [6]. In this case, the peptide bonds formed by Tyr80 and Lys92 residues located at the “bottom” entrance into the Len c 3 internal cavity were cleaved with the formation of the large fragment 1–80 and the protein without C-terminal Phe93 (Figure 5, Figures S1 and S2 and Table S2). It is worth noting that the removal of Phe93 from Len c 3 by plant proteases occurs in lentil tissues [20]. The formation of the large fragment without C-terminal part has also been shown for peach and wheat LTP [23], as well as for LTP from grape [24]. In the cases of peach and wheat, this was due to cleavage of the peptide bond formed by Tyr79. The fragment 1–80 of Len c 3 was quite stable, possibly due to the fact that it was stabilized by three disulfide bonds. Besides, Tyr80, Lys92, Lys33, and Lys34 located on the surface of H2 helix, as well as Leu62 located at the “top” entrance into the Len c 3 internal cavity, were the most exposed sites for gastroduodenal proteolysis.

Previously, it has been shown that the major linear IgE-binding epitopes of the Pru p 3 allergen were located in the protein regions between 11–25, 31–45, 71–90 amino acid residues (Figure 5) [25,26]. Moreover, it has been demonstrated that the fragment of the grape LTP without the C-terminal part retained its IgE-binding, and the digested grape allergen kept its ability to stimulate basophil histamine release [24]. In our study, the rLen c 3 gastroduodenal digest (gastric and subsequent duodenal digestion for 2 h) showed remarkably lower capacity to bind sIgE from sera of patients with food and pollen allergy (Figure 6). In this case, rLen c 3 was degraded by 83%, but its fragment 1–80 containing a shortened C-terminal epitope was present in hydrolysate in meaningful quantity (Figure S1). Therefore, it was assumed that the C-terminal peptide of Len c 3, having a high percentage of homology with the C-terminal epitope of Pru p 3, played an important role in the ability of the lentil allergen to bind cross-reactive IgE.

**Figure 8:**
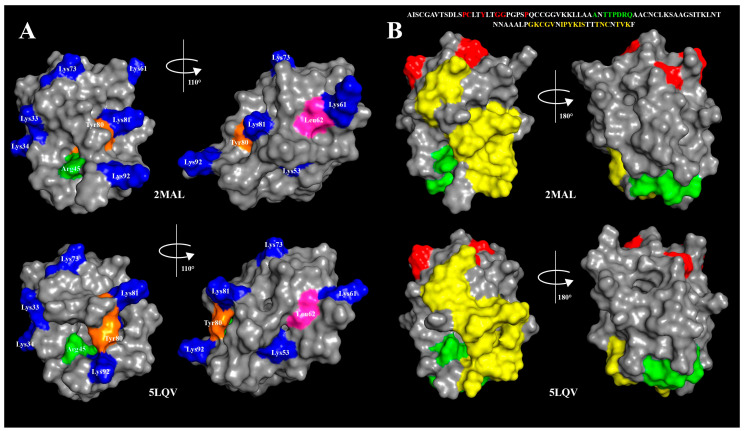
The NMR solution structures of the ligand-free Len c 3 (2MAL) and the Len c 3-LPPG complex (5LQV). (**A**) Key amino acid residues subjected to cleavage: lysines are colored in blue, Arg45 in green, Tyr80 in orange, and Leu62 in magenta. (**B**) The residues forming three linear epitopes of Pru p 3 are colored in red, green, and yellow.

At the next stage, effects of lipid ligands on rLen c 3 digestion were examined. Previously, it has been shown that lipid ligands can increase (in the case of the wheat LTP [23]) or decrease (in the case of the grape LTP [24] and Bet v 1 homolog Ara h 8 from peanut [27]) the rate of gastroduodenal digestion. In our work, it was shown that the presence of all lipid ligands except BEH reduced the rLen c 3 degradation rate, but the effect level depended on the nature of a ligand. These data correlated well with data on an influence of lipid ligands on the allergen thermostability. The most pronounced effect was also observed for LPPG. The rate decrease of the rLen c 3 degradation was slightly less pronounced for OLE. Earlier, we showed that the volume of the Len c 3 hydrophobic cavity increased upon LPPG binding, but the spatial structure of the protein changed not so much (PDB ID: 5LQV) [8]. Location of the key amino acid residues subjected to cleavage slightly changed upon the Len c 3-LPPG complex formation (Figure 8A). However, many of them, for instance, Leu11, Leu15, Leu18, Leu36, Leu52, Lys53, and Leu62, were involved in formation of hydrophobic and van der Waals contacts with LPPG and thereby stabilized the protein–ligand complex. Apparently due to this, the ligand-bound allergen stability to digestion was increased. At the same time, accessibility of key amino acids in the ligand-bound protein was strongly increased in the case of Tyr80 and Arg45 and slightly decreased for Leu62 (Figure 8A). In accordance with this, the peptide fragments formed by the rLen c 3 digestion in the presence or without LPPG were almost the same. However, cleavage of the peptide bonds formed by carboxyl groups of Arg45 and Leu62 in the Len c 3-LPPG complex was more effective in the former case and less effective in the latter case, respectively, than in the ligand-free protein (Figure 5 and Table S2).

Finally, a study of IgE-reactivity in the cases of the intact or preincubated with lipid ligands rLen c 3 was performed. Recently, it has been shown that preincubation of the peach allergen Pru p 3 [28], the walnut Jug r 3 [29], the apple Mal d 3, and the hazelnut Cor a 8 [30] with OLE increased IgE-binding capacity of the allergens. It has been assumed that changes in protein structures upon complex formation with this ligand could lead to an exposure of additional IgE epitopes. Such modifications were not observed in the case of the olive pollen allergen Ole e 7. IgE-binding capacity of this allergen in the presence of different lipid ligands or without them was the same [31]. In our experiments with the sera of patients with food and pollen allergy, we investigated an influence of two FAs, namely OLE and BEH, and two lysolipids (Figure 7). A slight increase in IgE-reactivity was found only for rLen c 3 preincubated with LPPG. This effect could occur due to increase in the availability of the C-terminal linear epitope in the structure of LPPG-bound protein (Figure 8B). Two other linear epitopes did not change their conformation upon binding with LPPG.

## 5. Conclusions

It is known that food allergy is often accompanied by various diseases of the gastrointestinal tract, manifested in changes of pH and production of digestive enzymes, inflammation, and increase of permeability of the epithelium. All of this leads to an impaired digestion, intestinal uptake of food proteins, and increase of their ability to cause allergic reactions. Recent studies showed that lipid ligands could also affect physicochemical and immunogenic features of proteins [4]. In our study, we showed that the lentil Len c 3 food allergen was sensitive to heating and digestion, but the presence of its possible endo- and exogenic lipid ligand might increase the protein thermostability during food cooking, decrease the rate of its gastroduodenal degradation, and, as a result, retain its IgE-binding capacity. Moreover, an extent of these effects depends on the nature of lipid ligand. Thus, based on our present results, it becomes clear that the sensitization capacity of ligand-binding allergens is linked with their lipid environment. Thus, the allergenic capacity of Len c 3 is largely dependent upon cooking and eating with other food possibly containing acceptable lipid ligands.

## Figures and Tables

**Table 1:** Comparative ligand binding characteristics of rLen c 3 at pH 7.4 and 2.5.

Ligand	IC_50_ (μM) *	
	10 mM Phosphate Buffer, pH 7.4	150 mM Sodium Chloride, pH 2.5
LAU	2.1 ± 0.2	2.1 ± 0.01
STE	4.5 ± 0.1	5.1 ± 0.1
OLE	2.4 ± 0.1	2.6 ± 0.1
BEH	5.9 ± 0.2	6.2 ± 0.1
LPPC	7.7 ± 0.02	7.3 ± 0.01
LPPG	4.3 ± 0.2	4.3 ± 0.03

* IC_50_ values were determined by competitive displacement of lipid ligands with 2-*p*-toluidinonaphthalene-6-sulphonate (TNS).

## Supplementary Materials

The following supplements are available online at https://www.mdpi.com/2218-273X/10/12/1668/s1. Figure S1: Analysis of duodenal allergen degradation. Figure S2: MALDI mass spectra of the RP-HPLC fractions (27, 28 and 29 min) of gastroduodenal digests of rLen c 3 alone and in the presence of LPPG. Table S1: Secondary structure estimation (%) predicted from Far-UV CD spectra. Table S2: MALDI mass analysis of the gastroduodenal digests (gastric and subsequent duodenal digestion for 2 h) of rLen c 3 alone and in the presence of LPPG. Table S3: Characterization of patient sera containing sIgE to rLen c 3.

## Abbreviations

LPPC	lyso-palmitoyl phosphatidylcholine
LPPG	lyso-palmitoyl phosphatidylglycerol
FA	fatty acid
LAU	lauric acid
STE	stearic acid
BEH	behenic (docosanoic) acid
OLE	oleic acid
TNS	2-*p*-toluidinonaphthalene-6-sulphonate
LTP	lipid transfer protein
TFA	trifluoroacetic acid
