# Supplementary material for: Impact of Different Lipid Ligands on the Stability and IgE-Binding Capacity of the Lentil Allergen Len c 3
Source: Biomolecules. 2020 Dec 13;10(12):1668. doi: 10.3390/biom10121668 (PMC7763088; doi:10.3390/biom10121668)
Supplement: Supplementary file 1 [file biomolecules-10-01668-s001.pdf]

Supporting information

Materials and Methods

Production of the recombinant allergens

The recombinant allergens were expressed as thioredoxin fusion proteins under control of the T7 promoter in *E. coli*. The BL-21 (DE3) cells transformed with pET-His8-TrxL-Len c 3 or pET-His8-TrxL-Pru p 3 were grown at 37°C in LB medium containing 100 mkg/mL of ampicillin and 20 mM glucose up to OD_{600} 0.6-0.7. Then the cells were induced by adding 0.2 mM isopropylthio-β-D-galactopyranoside and further were incubated at 200 rpm for 4–12 h. Incubation temperature was lowered down to 30°C. After incubation the cells were harvested by centrifugation at 4,000 *g* and lysed by sonication on ice in the binding buffer (50 mM Tris-HCl, pH 7.8, 0.5 M NaCl, 20 mM imidazole) containing 1 mM phenylmethylsulfonyl fluoride. The soluble fractions were separated by centrifugation at 25,000 *g* for 20 min at 4°C and loaded onto a column with Ni Sepharose. The bound proteins were eluted with the binding buffer containing 0.5 M imidazole. After overnight dialysis against 0.15% acetic acid in water, the lyophilized fusion proteins were dissolved in 80% TFA (10 mg/mL) containing an equal mass of cyanogen bromide. The reaction mixtures were incubated for 16 h at 20°C in the dark. After 10-fold dilution with water, the samples were lyophilized and then subjected to a round of subtractive metal chelate chromatography under denaturing conditions (in the same buffer system containing 6 M guanidine hydrochloride). RP-HPLC was performed on a Reprosil-Pur C18–AQ RP column (5 μm, 250×10 mm, Dr. Masch, Ammerbuch, Germany) using a linear gradient from 5 to 80% acetonitrile with 0.1% TFA for 60 min at a flow rate of 2 mL/min. Homogeneity and identity of the recombinant allergens were confirmed by SDS-PAGE, MALDI mass spectrometry, CD spectroscopy and N-terminal automated microsequensing (Procise cLC 491 Protein Sequencing System, PE Applied Biosystems, Foster City, California, USA).

Table S1. Secondary structure estimation (%) predicted from Far-UV CD spectra.

Sample	Conditions	〈-helix, %	®-sheet, %	turn, %	random, %	NRMSD*
Len c 3	20 °C	28.4	19.1	22.1	30.3	0.01
	98.5 °C	6.6	37.4	22.0	33.9	0.03
	20 °C end	5.6	37.7	21.8	34.9	0.04
Len c 3 +LPPG	20 °C	31.7	17.0	21.8	29.5	0.01
	98.5 °C	23.8	23.6	22.2	30.3	0.01
	20 °C end	31	17.3	22.2	29.4	0.01
Len c 3 +LPPC	20 °C	41.7	12.0	17.8	28.5	0.02
	98.5 °C	18.1	27.0	22.4	32.5	0.03
	20 °C end	9.4	34.7	22.1	33.7	0.02
Len c 3 +LAU	20 °C	26.4	21.2	21.8	30.7	0.01
	98.5 °C	14.5	33.5	21.1	30.8	0.02
	20 °C end	15.8	31.6	20.6	32.0	0.02
Len c 3 +STE	20 °C	26.5	22.5	21.0	30.1	0.02
	98.5 °C	8.6	35.6	22.5	33.3	0.04
	20 °C end	17.8	29.4	20.6	32.3	0.02
Len c 3 +OLE	20 °C	29.1	18.4	22.0	30.5	0.01
	98.5 °C	16.2	30.1	22.5	31.1	0.04
	20 °C end	20.1	25.6	22.3	32.1	0.02
Len c 3	20 °C	32.0	16.2	22.1	29.8	0.01

+BEH	98.5 °C	14.9	29.1	23.4	32.5	0.02
	20 °C end	15.0	30.7	22.1	32.2	0.02

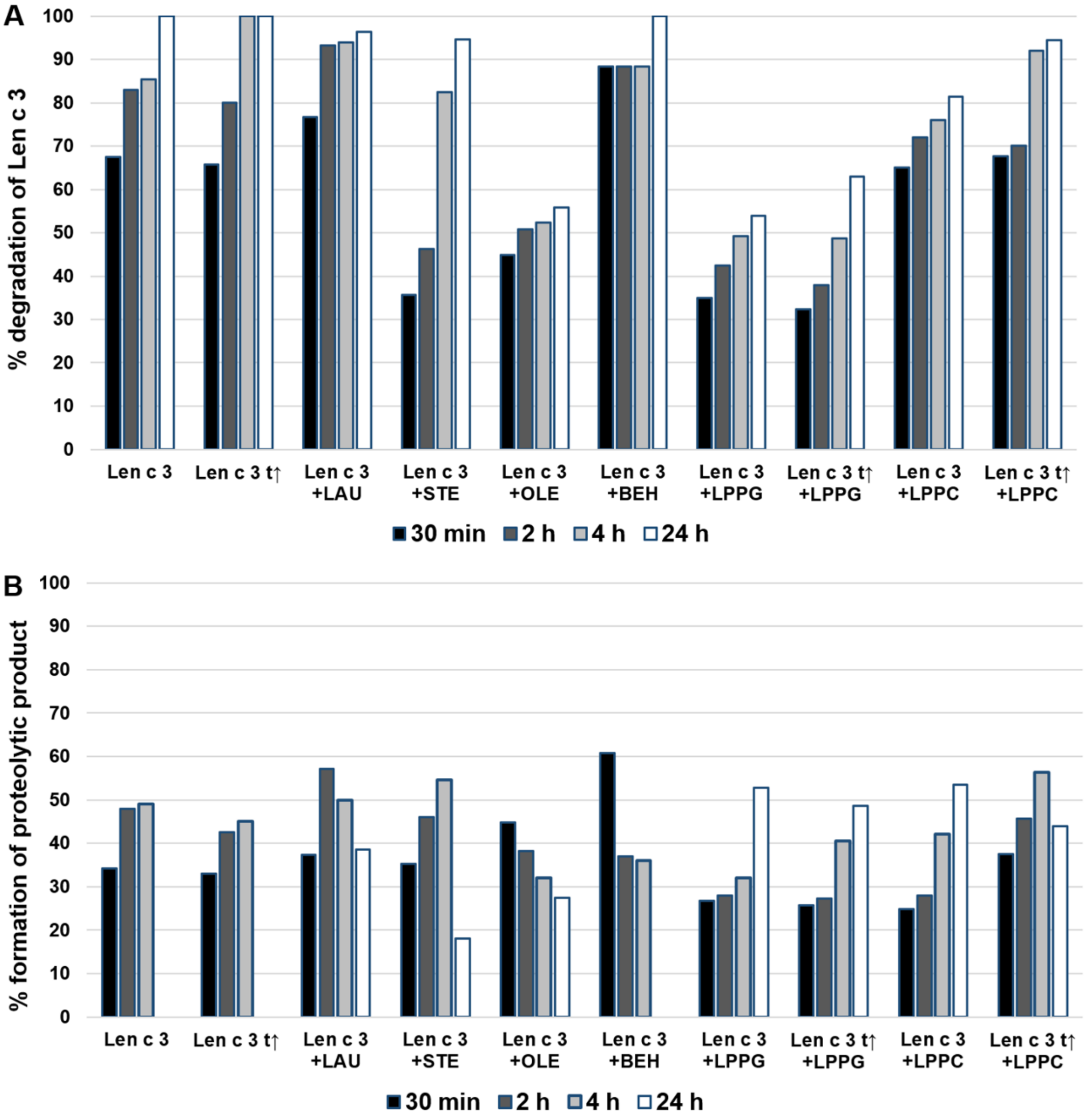

Figure S1. Analysis of duodenal allergen degradation. The bands corresponding to intact protein (A) and proteolytic product with M.w. 6-8 kDa (B) were scanned using laser densitometer. % Degradation of rLen c 3 was calculated as (So-Sa)/So*100%, where So is the area of rLen c 3 band at the initial time (0 min) and Sa is the area of allergen band at the studied time. % Formation of proteolytic product was calculated as (Sp)/So*100%, where Sp is the area of proteolytic product band at the studied time. Analysis of gastric protein digestion is not given since the allergen is practically not cleaved by pepsin.

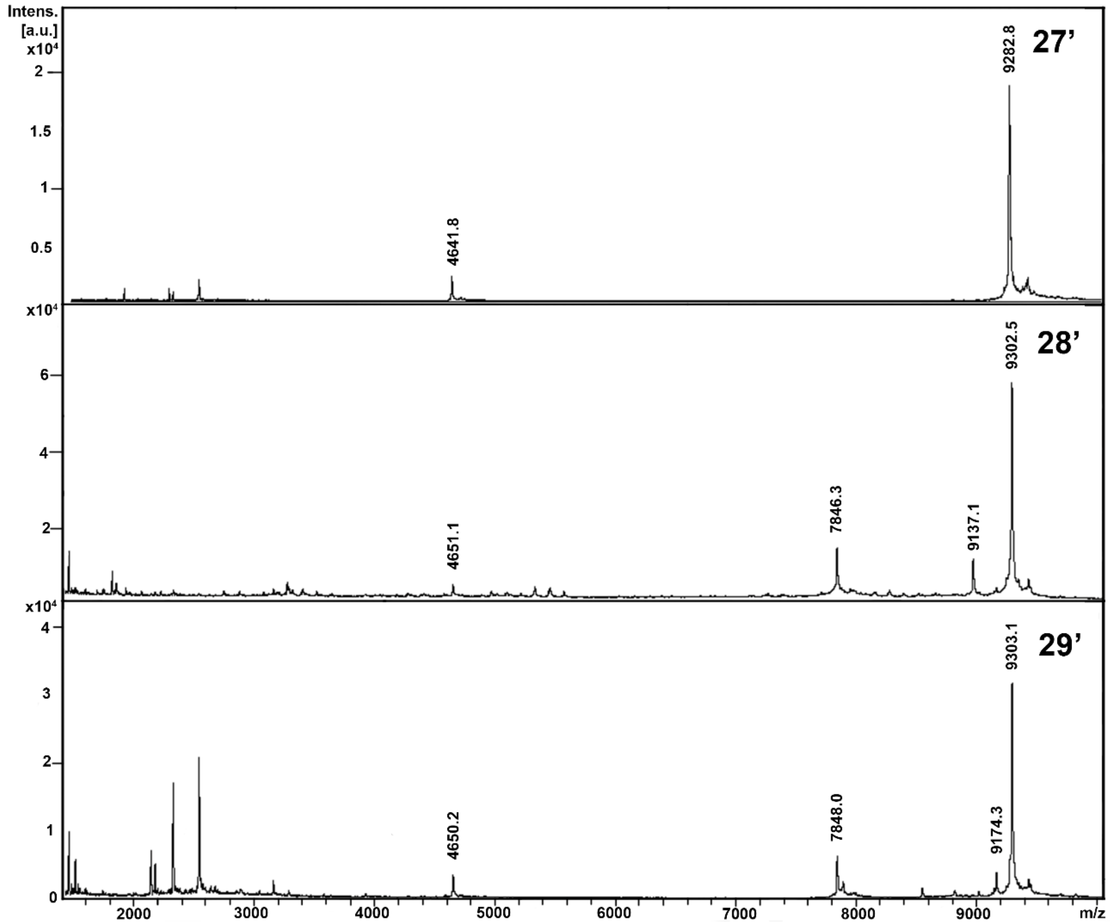

Figure S2. MALDI mass spectra of the RP-HPLC fractions (27, 28 and 29 minutes) of gastroduodenal digests of rLen c 3 alone and in the presence of LPPG.

Table S2. MALDI mass analysis of the gastroduodenal digests (gastric and subsequent duodenal digestion for 2 hours) of rLen c 3 alone and in the presence of LPPG.

Fragment	Calculated mass $[M+H]^+$ (Cys is oxidized)	Observed mass $[M+H]^+$		Number of Cys
		Len c 3	Len c 3+LPPG	
1-15	1435.7	1436.9	1436.9	2
1-17	1699.9	-	1702.0	2
1-18	1813.1	-	1815.9	2
1-33 with cleavage of peptide bond	3155.6	3156.8	3156.8	4
1-34	3265.8	3270.6	3269.9	4
1-34 with cleavage of peptide bond	3283.8	3284.7	3284.5	4
1-80	7846.0	7846.9	7847.7	7
1-92	9136.5	9137.4	9135.8	8
1-93	9283.6	9284.8	9284.8	8
1-93 with cleavage of peptide bond	9301.6	9302.7	9302.7	8
16-33	1720.9	1720.9	1720.9	2
18-34	1584.9	1587.0	1587.0	2
19-33	1343.5	1343.9	1344.2	2
19-34	1471.7	1472.0	1471.9	2
34-45	1271.5	-	1271.6	-

35-45	1143.3	1143.0	1143.0	-
35-52	1845.1	1847.9	-	2
36-45	1030.1	-	1030.0	-
46-62	1677.9	1676.0	1675.9	2
54-62	847.9	847.2	847.0	-
62-73	1184.3	-	1184.0	-
63-73	1071.2	1071.0	1071.0	-
63-80	1817.1	1817.9	-	1
63-93	3254.7	3254.4	-	2
81-92	1309.5	1310.1	1310.0	1
81-93	1456.7	1457.0	1456.9	1

Table S3. Characterization of patient sera* containing sIgE to rLen c 3.

No.	Age (y.o.)	Sex (M/F)	sIgE to peanut		Highest sIgE level			Other allergen sources
			IU/ml	RAST class	Source	IU/ml	RAST class	
1	4	F	8.03	3.3	birch	>100	6	po, hz
2	4	F	2.68	2.7	peanut	2.68	2.7	B
3	42	M	0.66	1.8	-	-	-	nd
4	6	M	1.23	2.1	grass	5.86	3.1	B, wt, hz
5	3	M	0.43	1.2	wheat	0.89	2	nd
6	3	M	1.01	2.1	birch	>100	6	A, H, G, R, hz, ct, wt
7	2	M	0.84	2	birch	2.2	2.5	nd
8	3	M	1.9	2.4	hazelnut	49.53	4.9	B, G, R, M, sb
9	4	F	0.74	2	wheat	8.3	3.3	nd
10	6	M	2.17	2.5	rye	5.94	3.1	B, hz, wt
11	3	M	4,52	3	birch	>100	6	hz, ct, wt
12	3	M	8.01	3.3	birch	>100	6	hz, wt
13	15	M	0.51	1.4	-	-	-	nd
14	1	M	8.3	3.3	peanut	8.3	3.3	hz
15	4	F	0.49	1.4	wheat	0.62	1.7	nd
16	2	F	1.23	2.1	hazelnut	2.62	2.6	wn, cl
17	1	F	4.39	3	hazelnut	25.96	4.2	am, wt, ry
18	11	M	1.85	2.4	birch	>100	6	G, wt, hz, ct
19	10	M	0.19	0.5	peanut	0.19	0.5	nd

20	2	F	>100	6	peanut	>100	6	hz, sb, po, wt
21	7	M	3.68	3	birch	>100	6	A, H, R, hz, ct, wt, sb
22	3	M	2.68	2.7	birch	>100	6	G, sb, ct, po, wt, hz
23	4	M	0.89	2	birch	>100	6	G, hz
24	4	F	0.49	1.4	birch	3.52	3	G, ct, po, wt
25	6	M	0.55	1.5	rye	18.57	4	ct, ap, wt
26	2	F	0.32	0.9	birch	15.71	3.8	nd
27	2	M	1.06	2.1	wheat	2.17	2.5	nd
28	3	F	0.89	2	peanut	0.89	2	nd
29	3	F	>100	6	birch	>100	6	A, H, hz
30	7	F	-	-	birch	17.93	4	A, H, R
31	15	F	-	-	celery	0.95	2	po
32	4	M	-	-	birch	7.79	3.3	A, H, G, R, M
33	25	M	0.7	2	mugwort	48.9	4.9	G, R, sb, wt, hz, wn, cl, ap
34	4	M	-	-	mugwort	>100	6	G, R
35	10	F	-	-	birch	>100	6	A, H
36	11	M	6.32	3.2	birch	>100	6	A, H, G, R, M, sb, ct, wt, hz
37	11	M	-	-	birch	>100	6	A, H, R, G
38	4	M	3.7	3.7	birch	94.09	5.8	G, sb, ct, po, wt, hz

nd – not determined. *Patients with food and pollen allergy had such allergic symptoms as rhinitis, conjunctivitis, atopic dermatitis, and oral allergy syndrome.

Description of RAST classes: 0 class – not detectable or trace IgE level [0,00-0,34 IU/ml]; 1 class – threshold level [0,35-0,69 IU/ml]; 2 class – elevated IgE level [0,70-3,49 IU/ml]; 3 class - significantly elevated IgE level [3,50-17,49 IU/ml]; 4 class – high IgE level [17,5-49,99 IU/ml]; 5 class – very high IgE level [50,0-99,99 IU/ml]; 6 class - extremely high IgE level [⩾100,0 IU/ml]

Inhalant: B = birch pollen, G = grass pollen, A = alder pollen, H = hazel pollen, R = rye pollen, M = mugwort pollen

Food: sb = soybean, ct = carrot, po = potato, wt = wheat, hz = hazelnut, am = almond, wn = walnut, ry = rye, cl = celery, ap = apple
